# Urban Economies and Occupation Space: Can They Get “There” from “Here”?

**DOI:** 10.1371/journal.pone.0073676

**Published:** 2013-09-09

**Authors:** Rachata Muneepeerakul, José Lobo, Shade T. Shutters, Andrés Goméz-Liévano, Murad R. Qubbaj

**Affiliations:** 1 School of Sustainability, Arizona State University, Tempe, Arizona, United States of America; 2 Mathematical, Computational, and Modeling Sciences Center, Arizona State University, Tempe, Arizona, United States of America; 3 Center for Social Dynamics and Complexity, Arizona State University, Tempe, Arizona, United States of America; 4 School of Human Evolution and Social Change, Arizona State University, Tempe, Arizona, United States of America; MIT, United States of America

## Abstract

Much of the socioeconomic life in the United States occurs in its urban areas. While an urban economy is defined to a large extent by its network of occupational specializations, an examination of this important network is absent from the considerable body of work on the determinants of urban economic performance. Here we develop a structure-based analysis addressing how the network of interdependencies among occupational specializations affects the ease with which urban economies can transform themselves. While most occupational specializations exhibit positive relationships between one another, many exhibit negative ones, and the balance between the two partially explains the productivity of an urban economy. The current set of occupational specializations of an urban economy and its location in the occupation space constrain its future development paths. Important tradeoffs exist between different alternatives for altering an occupational specialization pattern, both at a single occupation and an entire occupational portfolio levels.

## Introduction

Much of the socioeconomic life in the United States occurs in its urban areas, or more precisely, in its almost 400 Metropolitan Statistical Areas (MSAs). These MSAs generate more than 90% percent of the country's output and house more than 84% of its population. These metropolitan economies, as with all modern economies, are intricate webs linking specialized production units [1]–[4]. What goods and services such units can provide, and how well they provide them, is largely determined by the technologies, skills, and tacit knowledge integrated in the process of value creation. The ease with which an economy can shift to new activities is largely determined by its current portfolio of technologies and skills [5]–[9]. It is intuitively compelling to hypothesize that the interconnections among these technologies and skills form an economic structure, enabling some developmental pathways while foreclosing others. Recent work by Hidalgo et al. [10] shows that such a structure is indeed crucial for understanding the economic development at the national level: the technologies and skills prevalent in the economy of a country, embodied in the goods it produces and services it provides, place that economy in a specific region of a global “product space” and constrain the ease with which that economy can transform its production structure.

Here we bring an explicit structural perspective to bear on the question of transformations of U.S. urban economies by analyzing occupational data at the MSA level (publicly available at http://www.bls.gov/oes). MSAs offer a propitious setting for exploring how the interconnections among economic activities channel transformative possibilities. Not only can capital, labor, and information flow freely among MSAs, they also share similar legal, political, linguistic, and cultural frameworks, thereby eliminating effects that often confound cross-national economic studies.

Occupations, as classified by the U.S. Bureau of Labor Statistics (BLS), are based on the work they carry out and the skills, education, training, and credentials needed to perform the work. Therefore, the occupational data capture not only the products and services, but also the skills that characterize urban economies. It is skills that better capture the human capital present in the labor force [11]–[14], and human capital is a decisive determinant in the generation of innovations and the development of new industries in urban economies [15], [16].

Here we employ the occupational data to investigate the extent to which the set of interacting specialized skills of an MSA's economy, and its place in an “occupation space,” constrain the ease with which the MSA can transform itself. To construct a metropolitan occupation space we require a “distance” between any given pair of occupations. Here we derive such distance from a novel metric based on occupational specialization patterns in the MSAs. What makes an MSA economically distinct are the occupations in which it specializes relative to others: think finance in the case of New York City, computer hardware and software in Silicon Valley, aerospace manufacturing in Seattle, and higher education in Boston [17]. To formulate our metric, we start with the traditional location quotient [18] of occupation 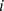 in MSA 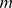, 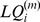, which is defined as:
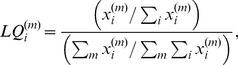
(1)where 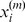 is the number of employees engaged in occupation 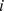 in MSA 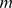. When 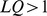 for an occupation in a given MSA, it means that the occupation is “overrepresented” in that MSA; that is, the proportion of the MSA's labor force engaged in that occupation is greater than that of its national counterpart, i.e., when all MSAs are considered. This indicates the MSA's specialization in that occupation, presumably due to some underlying location-specific conditions favorable for the occupation, such as labor force skills and availability, organizational and physical infrastructure, geographical attributes, natural endowments, and historical contingency.

## Results

### Interdependencies between occupational specializations

Our first task is to identify and quantify interactions among occupational specializations across MSAs. This task is made difficult by the absence of readily available data on material, personnel, financial, or informational flows among work places of the sort that would directly signify interaction among workers in different work places. How, then, can one infer from the presence of specialized occupations in an MSA that their co-location is not merely accidental but indicative of possible causal interaction? Here we employ conditional probability: specifically in this context, if the presence of one specialized occupation in an MSA is partly determined by the presence of another specialized occupation, one would expect conditional probabilities to differ from marginal ones. Accordingly, we define the “interdependency” between two occupations 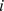 and 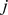, 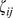, as:
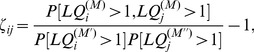
(2)where 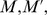 and 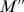 denote a randomly selected MSA (for brevity, the superscripts will hereinafter be omitted); see Methods for detailed discussion on 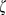. This metric measures how an MSA's specialization in one occupation *may* enhance or hinder its specialization in another. The emphasis on “may” acknowledges that – as is the case for many statistical analyses – without additional information or experiments, our analysis cannot imply direct causality; at best, it identifies structural relationships and points to potential places where one may search for such causality. With that caveat in mind, we now proceed to interpret 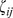. Positive 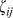 means that occupations 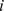 and 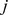 are more likely to be specialized in the same MSAs than if they are independently distributed across MSAs. The opposite is true for 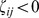, while 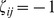 means that occupations 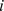 and 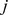 are never specialized in the same MSA. Clearly, 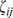 is closely tied to the more conventional way of representing conditional probability: 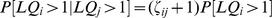. Note that 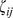 is intrinsically symmetric (i.e., 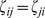) and thus a suitable metric for building an occupation space (see Methods), while the above relationship preserves the directionality of the conditional probabilities between two occupations, which will be important in our later analysis.

**Figure 1:**
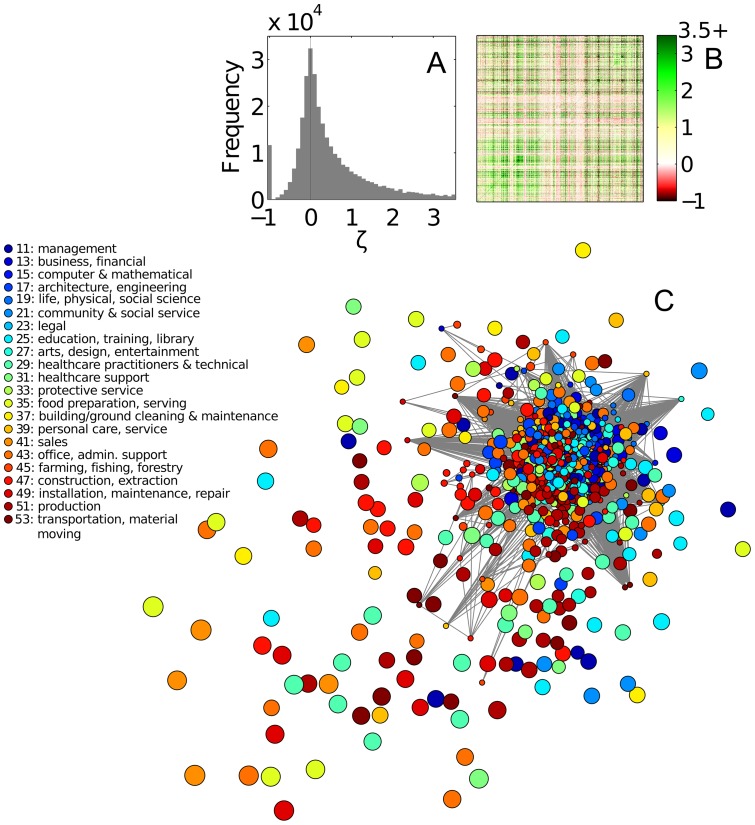
Interdependency 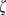 between occupations and the occupation space (2010 data). (A) the histogram of 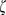; (B) 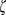 matrix between all 787 occupations; and (C) the 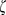-based occupation space. Some very large values of 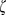 exist, typically resulting between uncommon occupations that are over-represented in the same MSAs; these are not shown in (A) and (B). In (C), each node represents an occupation code, the node color corresponds to one of the 22 2-digit occupation groups (as defined by BLS), and the node size depends on how many MSAs specialize in that occupation (i.e., 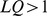). Large distances between occupations correspond to low or negative 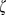, whereas short distances high positive 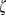 (see Methods). Only links corresponding to the highest (positive) 1% of 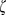 are included for figure's legibility.

We investigate the structure of occupational specializations in MSAs using 2010 data, the latest year for which both employment and GDP data were available at the MSA level. The 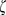 histogram (Fig. 1A) indicates that most occupations have positive interdependencies with one another, with 31.2% being negative and 3.8% equal to 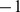. The figure also shows that most interdependencies are relatively weak, as indicated by the peak around zero; somewhat surprisingly, and notwithstanding the fact that cities are agglomerations of individuals and businesses, many occupational specializations within urban areas do not strongly interact with one another. Fig. 1B shows the 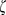 between all 787 distinct occupations considered in a matrix format. Occupations are simply ordered in accordance with BLS occupation grouping codes: the first 33 rows/columns of the matrix correspond to occupations whose codes start with 11 (management occupations); the next 30 rows/columns those starting with 13 (business and financial operations occupations); and so on. Consequently, the presence of some dark green areas (representing strong positive interdependencies) along the diagonal is to be expected: some occupations in the same classification group are closely related and, if an MSA specializes in one, it likely specializes in another. More interesting, however, are the white and red bands (weak or negative interdependencies) along the diagonal, and the off-diagonal dark green areas, indicating that strong positive interdependencies exist among occupations belonging to different occupational groups. These strong interdependencies define the structure of the occupation space.

### Structure of the occupation space

Deeper insights about the occupation space are gained by revealing its structure through the use of a network representation, shown in Fig. 1C (not all links are shown). Here the nodes represent occupations and the length of the edges between them represent their interdependencies. Several patterns emerge from this network. A number of occupations are specialized in only a few MSAs (small nodes). Many of these rarer specialties are specialized in the same MSAs, resulting in strong positive interdependencies and forming the “core” of the occupation space (Fig. 1C and also Fig. S6 in Supplementary Information S1). We also find that common occupations (larger nodes) interact relatively weakly with other occupations (i.e., they are specialized in many places regardless of what happens with other occupations (Fig. S2 in Supplementary Information S1)) and consequently are located along the periphery of the occupation space. Overall, these occupational specializations form a rather dense network of strong positive interdependencies (see Figs. S3, S4, and S5 in Supplementary Information S1): even when one considers only edges associated with 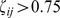, occupations still have, on average, hundreds of such strong interdependencies with other occupations with a high degree of clustering (Table S1 in Supplementary Information S1). It is worth noting that educational occupations are among those with the largest numbers of such strong positive links, highlighting their importance in urban economies (Table S1 in Supplementary Information S1). Additional topological features of the occupation space are also reported in SI.

**Figure 2:**
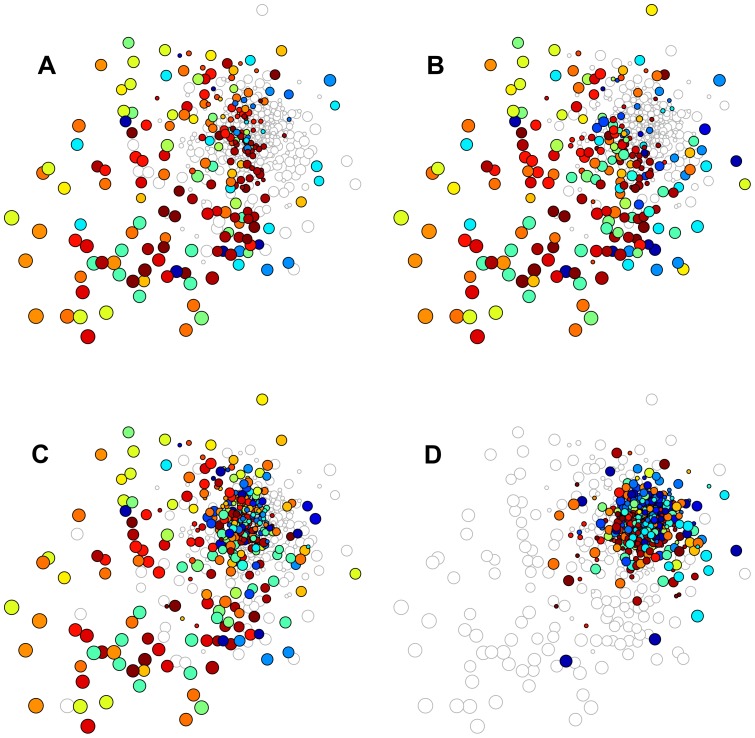
Specialized occupation sets (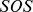) of MSAs belonging to different wealth classes. Specialized occupation sets of 4 classes of MSAs, categorized by their per capita gross domestic products (GDPs). (A) bottom class (first quartile); (B) lower-middle (second quartile); (C) upper-middle class (third quartile); and (D) top class (fourth quartile). For each class, employees in each occupation are summed across MSAs within that class. 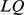
s are then calculated, essentially treating the class as if it is one ``super MSA.” Note that these spaces are the same as in Fig. 1C but with links removed to avoid cluttered figures.

What region of occupation space do metropolitan areas with different levels of productivity and wealth inhabit? Answering this question is critical to understanding how an MSA's specialization portfolio is related to its economic performance. We categorize MSAs into quartiles according to their 2010 GDP per capita (Fig. S1 in Supplementary Information S1), a measure of both productivity and wealth, and consider their *specialized occupations set* (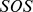), the set of occupations in which MSAs are specialized (i.e., 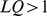). As one moves from the bottom to the top quartile, the 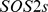 gravitate toward the occupation space's core region – characterized by uncommon occupations and strong positive interdependencies (Figs. 1C and 2). Wealthier MSAs specialize in more unique occupations than their poorer counterparts.

**Figure 3:**
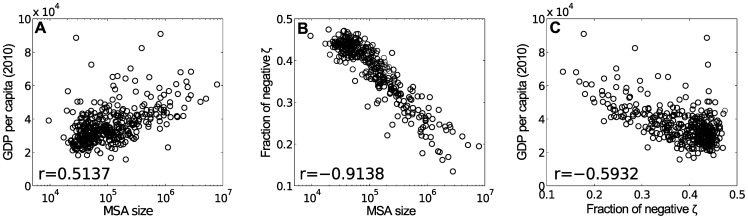
Size, GDP, and Interdependency. (A) MSA size–GDP per capita relationship; (B) MSA size–fraction of negative 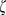 relationship; and (C) fraction of negative 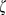
–GDP per capita relationship. The total number of employee is used as a proxy of the MSA size. Based on the correlation coefficients reported in the three panels, the partial correlation coefficient between fraction of negative 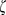 and GDP per capita with the MSA size held constant is 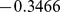.

No effect of the MSA size has so far been discussed. Size – in terms of population – has been shown to have strong relationships with a city's productivity, diversity, and specialization profile. How do the interdependencies among occupational specializations fit in these relationships? Fig. 3 shows the three-way relationship between size (for which the total number of employees being used as a proxy), GDP per capita, and fraction of interdependencies among specialized occupations that are negative. The partial correlation coefficient between the fraction of negative 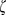 and GDP per capita with the MSA size held constant is 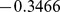: this represents the relationship between interdependencies and productivity with the effect of size filtered out. Together, Figs. 1, 2 and 3 suggest that on average larger cities are more productive, and their specialized occupations are more unique and have less negative interdependencies. Among cities of similar sizes, those with less negative interdependencies tend to have higher GDP per capita. An MSA's productivity thus depends not only on how many and what jobs are included in its 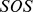 but also on the interdependencies among them.

### Constraints and tradeoffs in the occupation space

**Figure 4:**
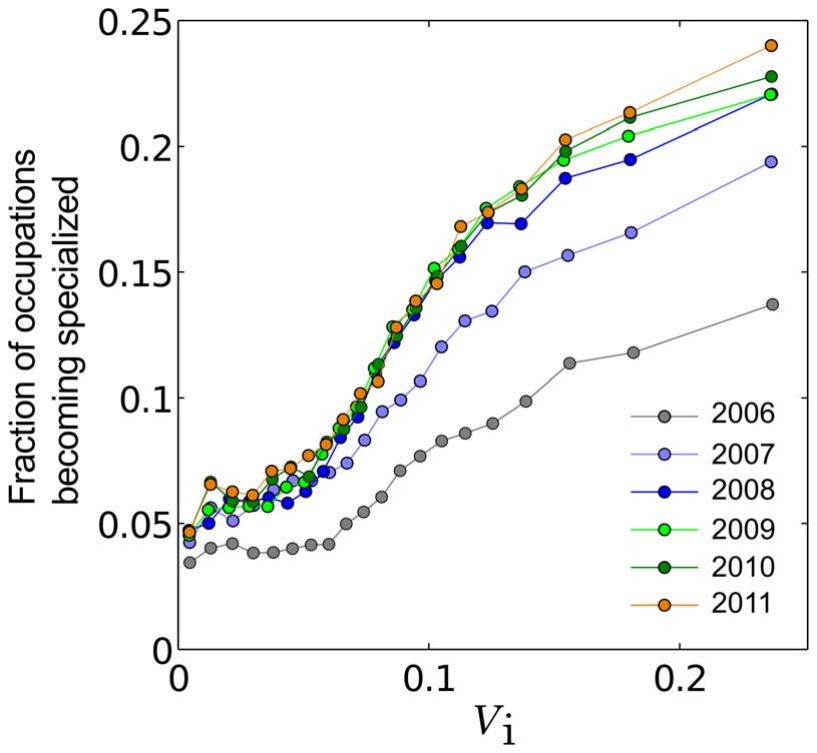
Constraints on changes in in occupation space. MSAs are more likely to specialize in occupations with more positive and less negative interdependencies with occupations in their current 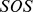.

In addition to its present productivity, the interdependency network within its 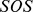 constrains an MSA's future economic trajectory. Intuitively, one might expect that an MSA is more likely to develop new specializations in occupations that have many positive interdependencies with occupations in its current 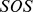. To quantify this notion, while capturing the effects of different signs and magnitudes of these interdependencies (cp. Ref. [10]), we introduce the transitional potential 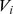 that a non-specialized occupation 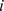 will become specialized in a later year:
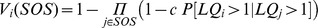


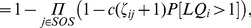
(3)where 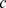 is a parameter (see Methods for additional discussion on Eq. 3). Fig. 4 shows that occupations with higher 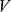 are indeed more likely to become specialized. (Keep in mind, though, that while an occupation with higher 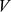 may potentially be specialized more easily, whether its specialization is efficient, desired by, or beneficial to the MSA is a different story (see Table S2 in SI); this issue is addressed in our next analysis.) Interestingly, the effects of such constraints seem to saturate after 3 years; this 3-year saturation pattern is robust even when different starting years are used (see SI).

**Figure 5:**
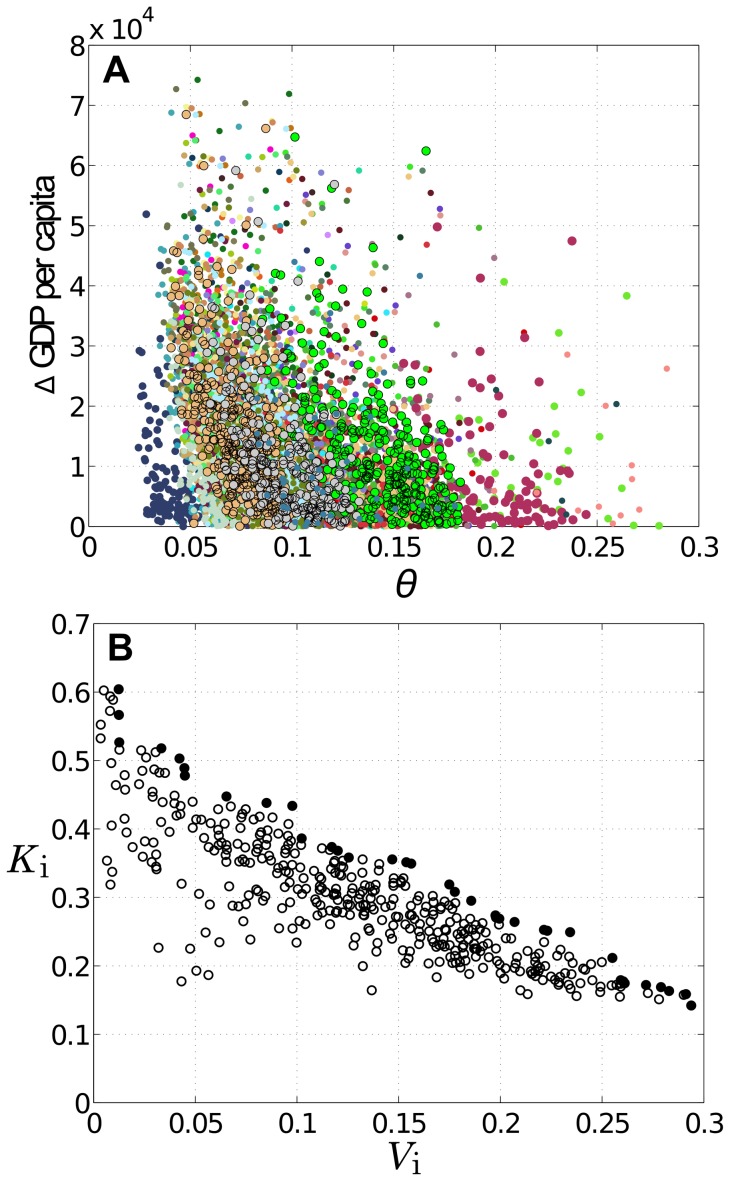
Tradeoffs on changes in occupation space. (A) relationship between ease of transformation from an MSA's 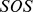 to that of another MSA and the corresponding difference/improvement in per capita GDP; and (B) relationship between the potential of the first transition and the potential of subsequent transformation (see text). In (A), 50 MSAs are randomly selected whose 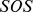
s are used as starting points; different colors specify different starting MSAs. In (B), the solid circles represent Pareto-efficient transitions.

How easy or difficult is it for an MSA to transform its 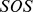
? Before proceeding, let us note for clarity that hereinafter “transition” refers to a change at the single-occupation level, whereas “transformation” refers to a change of an MSA's entire 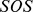; a transformation thus consists of many transitions. We define *transitional occupations* as those occupations with 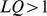 in a possible new 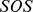 but with 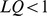 in the current (or original) set. Letting 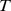 denote the set of transitional occupations, we write 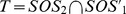, where subscripts 1 and 2 represent the original and the new 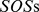, respectively, and the prime sign denotes a complement. We then measure the ease of transformation from one 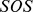 into another as the average of transitional potentials to all transitional occupations. Letting 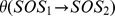 denote the ease of transformation for occupational portfolios 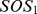 to 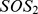, we write:
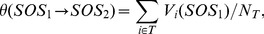
(4)where 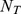 is the total number of transitional occupations. Note that 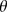 is asymmetric as it depends on the direction of transformation. Fig. 5A indicates that some tradeoff exists between the ease of transformation and the improvement of productivity that might result from it: more difficult transformations are generally associated with greater increases in metropolitan GDP per capita. As one might expect, there is significant uncertainty around this trend.

Given a set of transitional occupations associated with a transformation, what would constitute efficient pathways to achieve the transformation? What is the “best” first transitional occupation to pursue? Candidates for such an occupation should meet the following two properties: easy to transition to and conducive to further transitions. The first property is captured by the transitional potential 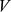. We propose to capture the second property by the average transitional probability from a given transitional occupation to all other transitional occupations, denoted by 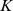; that is, for a transitional occupation 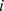,
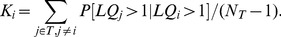
(5)


We apply our method to a specific example: transformation from the 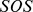 of the upper-middle quartile of MSAs (Fig. 2C), to that of the top quartile (Fig. 2D). The result is shown in Fig. 5B, which captures another type of tradeoff – between 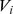 and 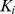. A Pareto frontier of the “optimal” transition occupations is observed: easier first transitions appear to be accompanied by more difficult subsequent ones. Equally useful is the analysis' ability to identify suboptimal transitions that may not be worth pursuing.

## Discussion

We show that a structural perspective augments our understanding of urban economies provided by the recent emphasis on occupational portfolios as a determinant of urban economic performance and a constraint for urban economic change. While co-located occupational specializations often interact positively with each other, many of them interact negatively. The balance between these interactions is then related to the size of an urban economy and partially explains its productivity and wealth. It is not only the set of current specialized occupations, but also the interdependencies among themselves and with the others in the occupation space, that determines an urban economy's possible development paths and how easy or difficult these paths will be. There also exist tradeoffs associated with changes in the occupation space both at the single occupation and the entire portfolio levels.

Our analysis on occupations complements existing research in economic geography focusing on industries [19]–[22] and technologies [23]. Occupations are not industry-based as many occupations are found across a number of industries. It is then possible that an industry may convert existing skills in an urban economy into occupations and create some new occupations with different skills. These occupations may in turn attract other industries, which in turn induce different sets of occupations, and so on in this “reciprocal spillover.” Uncovering and quantifying this kind of mechanism would reveal more deeply the workings of urban economies.

The present analysis offers tools for studying specialization, diversification, and growth processes of an urban economy – but they must be used with caveats and other considerations. For instance, we have used GDP per capita as a central measure in our analysis, i.e., dividing MSAs into classes and using it as a criterion of improvement. This was done for demonstrative purposes as GDP is a familiar, well-established metric. GDP, however, is not the only legitimate measure of an MSA: other concerns such as environmental quality, health, crime, literacy, and costs (direct and indirect) associated with the transitions can very well be taken into account. Inclusion of these additional dimensions would affect the choice of the desired occupational portfolio and result in – in place of the Pareto frontier in Fig. 5B – a more comprehensive Pareto ‘hyper-surface’; the notion of what the best transition is will of course change accordingly. Finally, this analysis, based on conditional probability, is general enough to incorporate other entities important to urban economies, such as industries and technologies, under one coherent framework; such integration promises to bring about deeper understanding of the workings of urban economics and warrants further investigation.

## Methods

### Location quotient

The values of 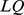 used in our analysis are calculated by applying Eq. 1 to the 364 MSAs considered (see Supporting Information for more details). Some reported values of 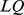 exist that may be based on employment data from both metropolitan and micropolitan statistical areas; these are not to be confused with the 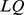
's in the present study.

### Interdependency 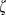


Our project was originally inspired by the work by Hidalgo *et al*. [10]. The key idea there was that what product an economy can specialize in is constrained by the economy's location in a “product space,” which represents how different products are related to one another. To Hidalgo *et al*., this suggests the notion of proximate and distant products, e.g., apples and pears have high proximity, as opposed to apples and copper wires [10]. This structural perspective is relevant to occupations in urban economies as well.

At the heart of their work is a measure that was introduced to capture the relationship between different products. The measure is called ‘proximity.’ Now if we were to apply proximity to our occupational data, the proximity between two occupations 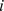 and 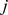 would be defined as:
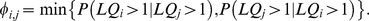



However, we found that there are a number of unsatisfactory properties associated with 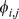.

First and most importantly, there are circumstances under which 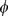, as a measure of relatedness between occupations, is ambiguous and misleading. The following counter-example illustrates this point. Consider two occupations 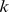 and 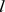 that are specialized in many MSAs (i.e., they are “common”) and are statistically independent of each other. The proximity between them is:
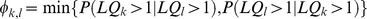


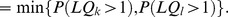



The second equality is obtained because, when considering two statistically independent events, the conditional probability is the same as the marginal probability. Then, because both are specialized in many MSAs, both 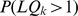 and 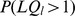 would be large. Thus, 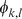 is large. These lead us to the following result: two statistically *independent* occupations can have *large proximity* – which implies a strong positive relationship – between them, and accordingly would appear close to each other in the network representation. Such an outcome is misleading.

Second, the symmetry of the proximity, i.e., 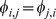, is not intrinsic, but is “forced” by the minimum operator. The symmetry is desired for building the network representation of the occupation space: the distance between two occupations should be based on only one number.

Third, there is no objective threshold of 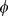 to determine which relationship is beneficial and which is not: it seems that a link, *regardless of its corresponding* 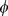, is always beneficial (see, e.g., the so-called “density” in Ref. [10]). We do not subscribe to this view, and believe that a relationship between two occupations can be either supportive or conflictive in nature.

It turns out that all these unsatisfactory properties can be eliminated by recognizing that conditional probability by itself does not completely capture the relationship between two occupations: it must be compared with the marginal probabilities of the two occupations. This recognition leads us to propose the ‘interdependency’ between occupations 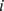 and 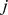 as follows:
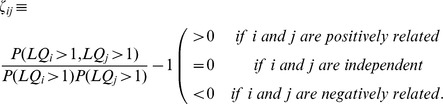



This new metric eliminates the ambiguity and misleadingness and logically captures the relationship between two occupations. It is intrinsically symmetric and can therefore be appropriately used to construct the network representation of the occupation space. The supportive or conflictive nature of the relationship can be easily identified by the sign of 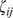.

### Network representation of occupation space

To construct the network representation of the occupation space, we use the forced-spring-and-repulsion scheme, in which the springs pull the nodes (occupations) closer together and the repulsion pushes them away. At the start, we assign very weak repulsion and very weak springs between all nodes. Then, for positive 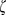
's, we add strength proportional to the magnitude of 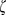
's to the springs. Similarly for negative 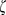
's, we add strength propositional to the magnitude of 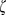
's to the repulsion. From the random initial conditions, the nodes are allowed to equilibrate with these pulls and pushes and eventually settle into a more-or-less steady-state configuration. This is what is shown in Fig. 1C. Note that it would take a very long time for the occupation space to reach the actual steady state, i.e., no node movements. What we present is the snapshot of the occupation space where the nodes are still moving, but at very, very low speeds, and the occupation space's topology and appearance shows essentially no change.

### Transitional potential

The expression of 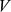, Eq. 3, is *motivated* by considering a successful transition of a previously non-specialized occupation 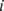 into a specialized one as a result of a decisive transition from one of the existing occupations in the MSA's current specialized occupation set (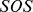) into occupation 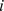. The probability of success of transitioning from occupation 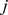 into occupation 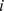 is assumed to be proportional to the corresponding conditional probability 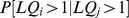 or, in terms of 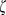, 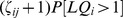. It is further assumed that each of these possible transitions is independent of one another. These considerations and assumptions lead the above expression, which is simply the probability that the transition from one or more currently specialized occupations are successful.

Note the emphasis on the term “motivated” in the previous paragraph. It serves as a reminder that we are *not* claiming that such independent transitions constitute the actual mechanism of transition from non-specialization to specialization of an occupation. As this work indicates, occupational specializations in urban economies form a complex network of interdependencies, and thus the actual mechanism of such a transition will surely be influenced by this network. Rather, the above expression of 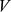 should be viewed as a kind of approximation in which higher-order interactions are excluded. Indeed, it is not at all uncommon that neglecting higher-order interactions allows one to construct useful and simple models or explanation of some complex phenomena (see, e.g., Ref. [24] for an example in neuroscience and Ref. [25] for another in ecology). In the present case, Figs. 4 and S7 in Supplementary Information S1 indicate that the formulation of 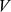 fulfills its purpose: a useful, theoretically-based measure of how an MSA's 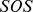 constrains its future occupational specializations.

In all the results presented in this paper, 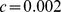 is used to calculate 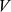. This value is simply chosen to result in a useful range of values of 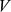: too high values of 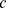 would yield 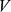
's that are always close to 1, while too low values of 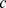 would yield 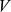
's that are always close to 0, both of which are not useful for our analysis. Finally, note also that for a very small 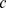, 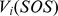 can be approximated by
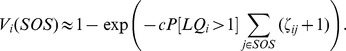



## Supporting Information

Supplementary Information S1 (PDF)
