# Supplementary material for: Urban Economies and Occupation Space: Can They Get “There” from “Here”?
Source: PLoS One. 2013 Sep 9;8(9):e73676. doi: 10.1371/journal.pone.0073676 (PMC3767603; doi:10.1371/journal.pone.0073676)
Supplement: Supplementary Information S1 — (PDF) [file pone.0073676.s001.pdf]

Urban Economies and Occupation Space: Can They Get "There" from "Here"?

Supplementary Information

Rachata Muneepeerakul,[1,2] José Lobo,[1]
Shade T. Shutters,[1,3] Andrés Goméz-Liévano,[2,4] & Murad R. Qubbaj[1]

[1]School of Sustainability,
[2]Mathematical, Computational, and Modeling Sciences Center,
[3]Center for Social Dynamics and Complexity,
[4]School of Human Evolution and Social Change,
Arizona State University, Tempe, Arizona 85287, USA

S1 Supplementary methods: notes on Metropolitan Statistical Areas (MSAs) and their occupational data

Our spatial unit of analysis in this study is the U.S. Metropolitan Statistical Area (MSA). An MSA is a geographical region centered around an urban core that includes adjacent counties having a high degree of social and economic integration with the core. MSAs are in effect unified labor markets. MSAs are defined by the U.S. Office of Management and Budget (OMB), and used by several Federal agencies for data collection and reporting purposes. However, the metropolitan definitions used by different agencies is not always consistent for a variety of reasons. To create the set of MSAs used in our analysis we had to reconcile the MSAs recognized by the Bureau of Labor Statistics (BLS), which reports on metropolitan occupations, and the set of MSAs used by the Bureau of Economic Affairs (BEA) when reporting on metropolitan Gross Domestic Product. Because our study included an analysis of data over the 2005-2011 period we removed eight MSAs that came into existence after 2005. Thus our set of metropolitan areas includes 364 MSAs whose 2010 employment data were used to build our occupation space.

Our final set of 787 occupations used to build occupation space was taken from 2010 BLS employment data at the MSA level (link "Metropolitan and nonmetropolitan area" in `http://www.bls.gov/oes/#data`). Like the MSA schema, the BLS periodically makes changes to its classification scheme for occupations. Though these changes are infrequent and typically minor, a new classification scheme was introduced by the BLS in 2005 that was so different from its previous scheme that comparisons between years before and after the change are not feasible. Therefore, we have limited our temporal analysis to years 2005 and later. A minor revision by the BLS took place in 2010, in which approximately 50 new occupation codes were introduced and a similar number of codes was discontinued. Fortunately, this change was essentially just a recoding of occupations and not a substantive change to the types of occupations that the BLS tracks. Therefore, a mapping of codes was straightforward and our occupational data from 2005-2010 are thus consistent and suitable for multi-year comparisons.

S2 Supplementary results and discussion

S2.1 Distribution of metropolitan GDP per capita

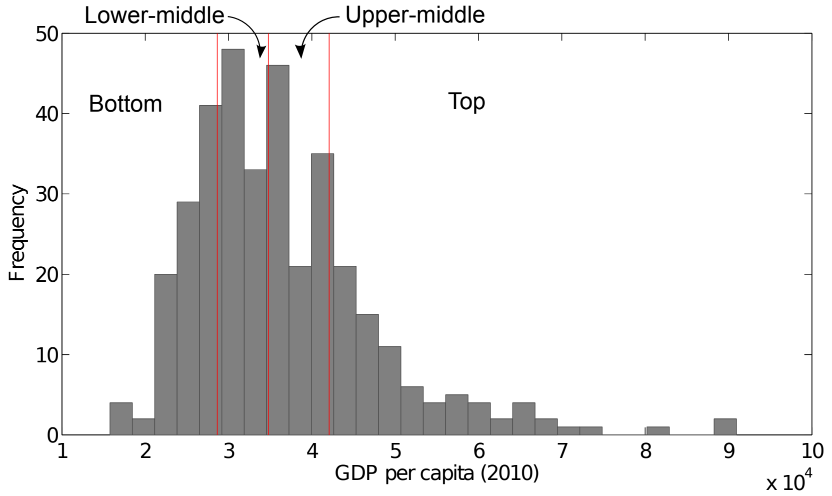

Figure S1: Histogram of the metropolitan GDP per capita and the four classes of MSAs used in the analysis in Fig. 2. The three red vertical lines represent the 25th, 50th, and 75th percentiles of the GDP per capita.

Fig. S1 shows the histogram of the GDP per capita of the MSAs. The MSAs are divided into four wealth classes—bottom (first quartile), lower-middle (second quartile), upper-middle (third quartile), and top (fourth quartile)—whose SOSs are located in the occupation space as shown in Fig. 2 in the main text.

S2.2 Commonness and interdependency

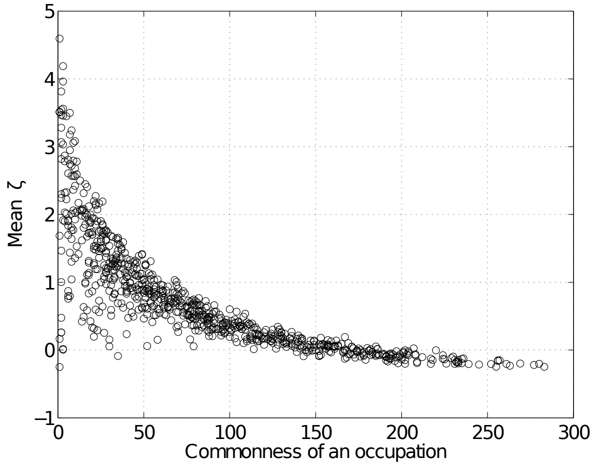

Figure S2: Relationship between "commonness" of an occupation, i.e., the number of MSAs that specialize in that occupation ($LQ > 1$) and the mean interdependency.

Fig. S2 is included here to support the following statement in the main text: "...common occupations (larger nodes), which tend to interact relatively weakly with other occupations (i.e., they are specialized in many places regardless of what happens with other occupations)." We also see that more unique or rare occupations tend to have strong positive interdependencies with other occupations, but with greater variations.

S2.3 Additional topological characteristics of the occupation space

Additional topological structure of the occupation space can be investigated by focusing on the relatively strong interdependencies, both positive and negative. Table S1 reports a number of standard network-theoretic metrics (namely degree and clustering coefficient) of the occupation space when only edges whose corresponding ζ's exceed a given threshold are included, and the remaining edges removed. (In the following discussion, the terms "edge" and "link" may be used interchangeably.)

Let us first focus on the strong **positive** interdependencies: these are occupations whose specializations tend to "support" one another. Clearly, as the threshold increases in either direction, the total number of edges, and thus degree, on average, decrease. What is somewhat surprising is that (as mentioned in the main text) even at a high strength threshold, the resulting network is still quite dense and clustered. For example, when considering only edges corresponding to $\zeta > 0.75$—given that an MSA specializes in one occupation, it is 75% more likely to specialize in another—the mean degree is still high at 230 with the mean clustering coefficient of 0.651. However, Fig. S3 shows that the distributions of the degree vary considerably for these different thresholds. At a low threshold of 0.10 (i.e., $\zeta > 0.10$), a majority of occupations are still highly connected with degrees between 400 and 600 or so. As the threshold increases, a large number of links are removed: this is consistent with our finding in Fig. 1A that most interdependencies are relatively weak. Importantly, at these large thresholds, the degree distributions become

Table S1: Additional topological characteristics of the occupation space, namely degree and clustering coefficient. Here, an edge is considered to exist only if its corresponding ζ exceeds a given threshold (in either positive or negative direction). A degree of an occupation is simply the number of such edges that it has. The occupations that form edges with an occupation is also called its neighbors. A clustering coefficient of an occupation is the ratio between the number of edges that actually exist and the number of all possible edges among its neighbors (*sensu* Ref. [1]).

Strong positive interdependencies, $\zeta > \alpha$

Threshold α	Occupation with maximum degree	Mean (Median) degree	Mean (Median) cluster. coeff.
0.10	Economics teachers, postsecondary	463 (547)	0.766 (0.800)
0.25	Engineering teachers, postsecondary	381 (469)	0.744 (0.777)
0.50	Atmospheric, earth, marine, and space sciences teachers, postsecondary	291 (352)	0.689 (0.748)
0.75	Anthropology and archeology teachers, postsecondary	230 (256)	0.651 (0.717)

Strong negative interdependencies, $\zeta < -\alpha$

Threshold α	Occupation with maximum degree	Mean (Median) degree	Mean (Median) cluster. coeff.
0.10	Log graders and scalers	168 (127)	0.217 (0.220)
0.25	Log graders and scalers	93 (66)	0.246 (0.216)
0.50	Log graders and scalers	43 (21)	0.539 (0.559)
0.75	Log graders and scalers	31 (14)	0.727 (0.800)

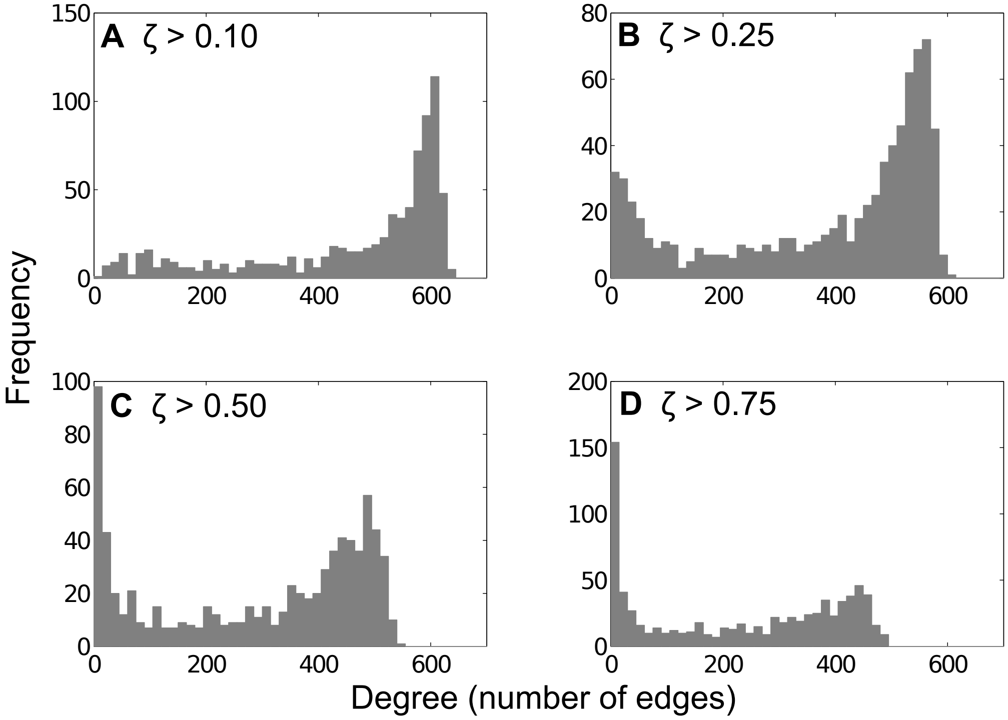

Figure S3: Degree distribution of strong positive interdependencies.

bimodal: the occupations can be topologically classified into two groups, those with high and low degrees (Figs. S3B, C, and D). The high-degree group represents those occupations whose specializations presumably require certain conditions that also benefit many other occupational specializations. These occupations form a tight cluster that is the core of the occupation space. Interestingly, when one considers the occupations with the maximum degrees for all thresholds (Table S1), one finds postsecondary teachers at these top positions, indicating the importance of educational occupations to urban economies.

Let us now focus on the strong **negative** interdependencies: these are occupations whose specializations tend to "hinder" one another. As the threshold increases in magnitude (i.e., only links with ζ's more negative than the threshold are included), the degree declines, but the distributions are consistently unimodal in this case (Fig. S4). Furthermore, at large negative thresholds, the links exhibit a rather tight clustering structure (see Table S1): most occupations within this group strongly hinder most other occupations. Recall that in the network representation, this translates to strong repulsion between these occupations.

All in all, the structure of the occupation space is determined largely by the strong positive interdependencies. Consider Fig. S5, the relationship between the number of links associated with $\zeta >$ 0.75 and $\zeta <$ -0.75 (also in conjunction with Figs. S3D and S4D). The figure shows that most occupations have similar degrees of strong negative interdependency, although a handful of them have remarkably many negative links. The difference in location of an occupation in the occupation space thus depends on the number of links with strong positive interdependencies it possesses. Fig. S6 illustrates this point.

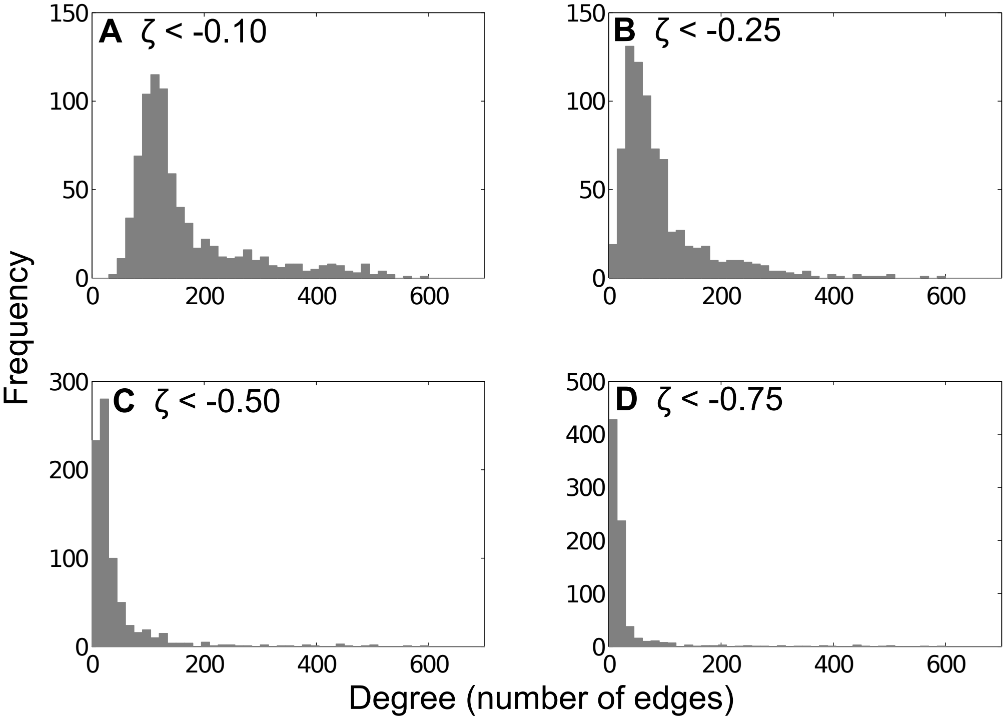

Figure S4: Degree distribution of strong negative interdependencies

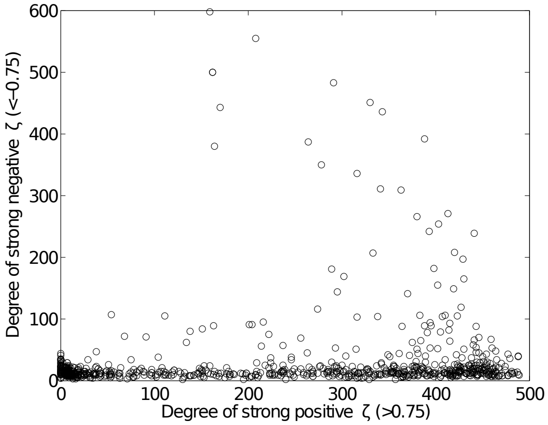

Figure S5: Relationship between degrees of strong positive and negative interdependencies. Each data point represents an occupation.

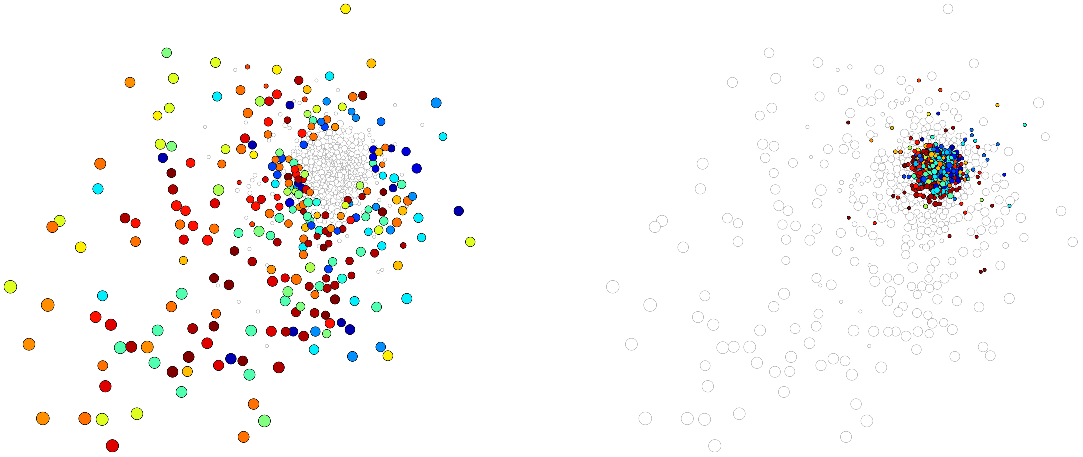

Figure S6: Effects of degree of positive interdependencies on the location of an occupation in the occupation space: (A) occupations with less than 100 links of $\zeta > 0.75$; and (B) occupations with more than 300 links of $\zeta > 0.75$.

S2.4 Current specializations and potential transitions in selected MSAs

In this section, we report the occupations with high and low location quotients (LQ's) and those with high and low transitional potentials (V's) in three MSAs spanning across the wealth spectrum. The occupations with high V's represent the occupations that are potentially easier for the MSA to transition into—whether any of those transitions will benefit the MSA is a different story: it may not lead to an increase in GDP; it may not be Pareto-efficient; or it may make subsequent transitions more difficult. To address this issue properly, a more subjective and comprehensive context is required. For example, in Fig. 5B, we use the SOS of the upper-middle class of MSAs as the starting point and that of the top class as the end point. This was done for demonstrative purposes. But GDP per capita is not the only legitimate measure of an MSA: other concerns such as environmental quality, health, crime, literacy, and costs (direct and indirect) associated with the transitions can very well be taken into account. Inclusion of these additional dimensions would affect the choice of the desired occupational portfolio and result in a Pareto 'hyper-surface,' as opposed to the Pareto frontier. And the notion of what the best transition is will of course change accordingly.

Table S2: Lists of occupations with 10 highest and lowest location quotients and occupations with 10 highest and lowest transitional potentials of three selected MSAs. Numbers in parentheses are 2010 GDP per capita.

10 highest LQ's	10 lowest LQ's	10 highest V's	10 lowest V's
**San Jose-Sunnyvale-Santa Clara, CA ($90,959)**			
Mathematical Science Occupations, All Other	Transportation Workers, All Other	Dental Hygienists	Continuous Mining Machine Operators
Semiconductor Processors	Crane and Tower Operators	First-Line Supervisors of Retail Sales Workers	Railroad Conductors and Yardmasters
Computer Hardware Engineers	Dredge Operators	First-Line Supervisors of Food Preparation and Serving Workers	Rail Transportation Workers, All Other
Computer and Information Research Scientists	Excavating and Loading Machine and Dragline Operators	Automotive Service Technicians and Mechanics	Manufactured Building and Mobile Home Installers
Software Developers, Systems Software	Hoist and Winch Operators	Nursing Aides, Orderlies, and Attendants*	Fallers
Sales Engineers	Gas Compressor and Gas Pumping Station Operators	Healthcare Support Workers, All Other*	Signal and Track Switch Repairers
Electronics Engineers, Except Computer	Pump Operators, Except Wellhead Pumpers	Cashiers	Roof Bolters, Mining
Aerospace Engineering and Operations Technicians	Wellhead Pumpers	Veterinarians	Mine Shuttle Car Operators
Choreographers	Mine Shuttle Car Operators	Pharmacy Technicians	Barbers
Tree Trimmers and Pruners	Tank Car, Truck, and Ship Loaders	Combined Food Preparation and Serving Workers, Including Fast Food	Log Graders and Scalers
**Phoenix-Mesa-Glendale, AZ ($41,169)**			
Signal and Track Switch Repairers	Ship Engineers	First-Line Supervisors of Food Preparation and Serving Workers	Railroad Conductors and Yardmasters
Environmental Science Teachers, Postsecondary	Bridge and Lock Tenders	Nursing Aides, Orderlies, and Attendants*	Rail Transportation Workers, All Other
Credit Authorizers, Checkers, and Clerks	Transportation Workers, All Other	Parts Salespersons	Manufactured Building and Mobile Home Installers
Flight Attendants	Dredge Operators	Cashiers	Fallers
Social Science Research Assistants	Hoist and Winch Operators	Combined Food Preparation and Serving Workers, Including Fast Food	Radio Operators
Forensic Science Technicians	Gas Compressor and Gas Pumping Station Operators	Registered Nurses*	Farm Labor Contractors
Airline Pilots, Copilots, and Flight Engineers	Pump Operators, Except Wellhead Pumpers	Dental Hygienists	Roof Bolters, Mining
Choreographers	Wellhead Pumpers	Healthcare Support Workers, All Other*	Mine Shuttle Car Operators
Credit Analysts	Mine Shuttle Car Operators	Pharmacists	Barbers
Recreational Vehicle Service Technicians	Tank Car, Truck, and Ship Loaders	Tellers	Log Graders and Scalers
**McAllen-Edinburg-Mission, TX ($15,775)**			
Graders and Sorters, Agricultural Products	Transportation Workers, All Other	Automotive Service Technicians and Mechanics	Continuous Mining Machine Operators
Agricultural Equipment Operators	Crane and Tower Operators	Registered Nurses*	Roof Bolters, Mining
Cleaning, Washing, and Metal Pickling Equipment Operators and Tenders	Dredge Operators	Pharmacists	Mine Shuttle Car Operators
Personal Care Aides	Hoist and Winch Operators	First-Line Supervisors of Mechanics, Installers, and Repairers	Railroad Conductors and Yardmasters
Audio-Visual and Multimedia Collections Specialists	Gas Compressor and Gas Pumping Station Operators	First-Line Supervisors of Production and Operating Workers	Rail Transportation Workers, All Other
Shoe and Leather Workers and Repairers	Pump Operators, Except Wellhead Pumpers	Light Truck or Delivery Services Drivers	Signal and Track Switch Repairers
Agricultural Inspectors	Wellhead Pumpers	Hotel, Motel, and Resort Desk Clerks	Locomotive Engineers
Service Unit Operators, Oil, Gas, and Mining	Mine Shuttle Car Operators	Bookkeeping, Accounting, and Auditing Clerks	Radio Operators
Counselors, All Other	Tank Car, Truck, and Ship Loaders	Dental Hygienists	Barbers
Agricultural and Food Science Technicians	Material Moving Workers, All Other	Waiters and Waitresses	Log Graders and Scalers

S2.5 Three-year saturation pattern

In this section, we report the results of the same analysis as that performed in Fig. 3B, with the only difference being the starting year. In Fig. 3B, we use 2005 as the starting year whose occupational data are used to calculate the transitional potential V of non-specialized occupations ($LQ < 1$); in Fig. S7, 2006 and 2007 are used instead. In all these plots, we observe that the influence of an MSA's specialized occupation set (SOS) on its future trajectory seems to saturate after 3 years. This indicates that the 3-year saturation pattern is not an artifact of using a particular year as the starting point.

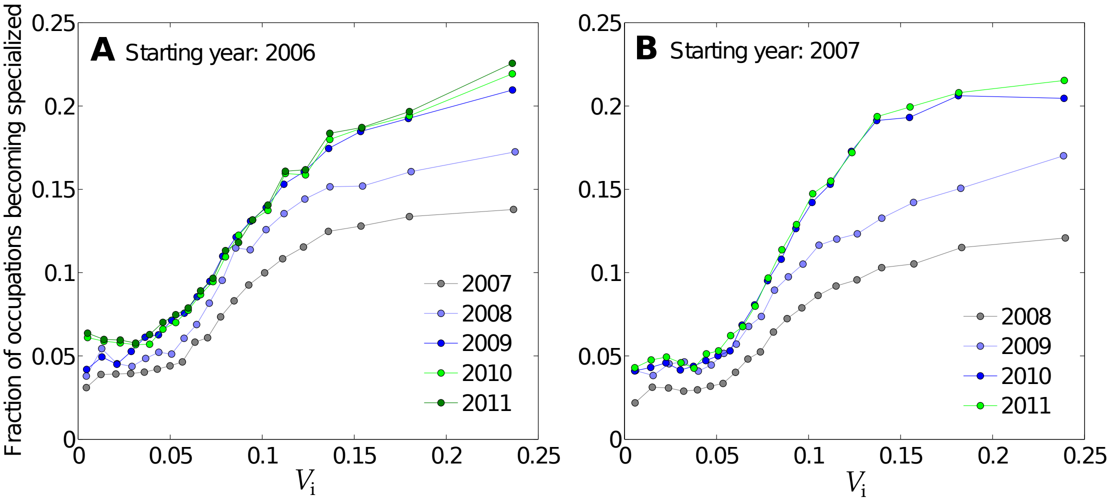

Figure S7: The proportion of occupations that become specialized given their transitional potential V: (A) 2006 as the starting year; and (B) 2007 as the starting year.

References

[1] Watts D, Strogatz S (1998) Collective dynamics of 'small-world' networks. Nature 393: 440-442.
